# Blue-Light Filters in Eye Care: A State-of-the-Art Review

**DOI:** 10.3390/medsci14050530

**Published:** 2026-08-30

**Authors:** Rowan Abass, Arwa Fathy, Ahmed Abass

**Affiliations:** 1 Department of Medicine, University of Cambridge, Cambridge CB2 0SP, UK; 2 Department of Engineering, University of Cambridge, Cambridge CB2 1PZ, UK; 3 Department of Materials, Design and Manufacturing Engineering, University of Liverpool, Liverpool L69 3GH, UK

**Keywords:** blue light, high-energy visible light, spectacle lenses, contact lenses, intraocular lenses, digital eye strain, sleep, circadian rhythm, retinal phototoxicity, commercial ophthalmic lenses

## Abstract

Blue-light filters now span spectacle, contact and intraocular lenses, but the term encompasses technologies with markedly different spectral characteristics and intended outcomes. This state-of-the-art narrative review synthesises peer-reviewed evidence on optical performance, digital eye strain, glare, photostress, sleep and circadian physiology, retinal phototoxicity, macular health, reported adverse effects and commercial translation. Near-clear spectacle filters preserve high-contrast acuity in most studies, but randomised and systematic reviews show no clinically meaningful average reduction in short-term digital eye strain. Some products reduce blue-colour contrast, particularly under low-contrast or low-light conditions, and adverse-event reporting is sparse. Studies of evening filters are heterogeneous: substantial amber attenuation can alter circadian light exposure, yet several trials have found no objective sleep benefit. Conventional visual outcomes with blue-filtering intraocular lenses are broadly comparable with ultraviolet-only lenses, although some individual studies report small mesopic colour or contrast disadvantages; long-term macular protection remains unresolved. Research on contact lenses is limited, typically short-term and often manufacturer-associated, and focuses on glare, photostress, scatter and perceived brightness rather than long-term health outcomes. Taken together, the evidence should be interpreted by filter spectrum and intended outcome, rather than treating blue-light filtering as a single intervention.

## 1. Introduction

Blue-light filtering has become a prominent theme across eye care, ranging from clear spectacle coatings, absorptive lens materials, strongly tinted evening eyewear, photochromic and fixed-filter contact lenses, and blue-filtering intraocular lenses (IOLs). The resulting literature is extensive but conceptually fragmented because the same label is applied to interventions that differ in wavelength cut-off, attenuation magnitude, luminous transmittance, activation state, wear schedule and anatomical position. Reviews consistently identify this heterogeneity as a central barrier to interpretation [1,2,3,4].

Interest has been driven by three broad propositions: that blue-light filtering may reduce symptoms associated with digital-device use; that it may influence sleep and circadian physiology; and that it may protect ocular tissues from photochemical injury. These propositions involve different exposure conditions and biological mechanisms. Evidence for one outcome therefore cannot be extrapolated automatically to another, and a statistically detectable laboratory effect does not necessarily translate into a clinically meaningful benefit in routine eye care [5,6,7,8,9,10,11,12,13,14,15].

The evidence base has also expanded beyond spectacles. Blue-filtering IOLs have been studied for their impact on visual function, sleep and macular health, while studies on contact lenses increasingly examine photochromic activation, high-energy-visible (HEV) attenuation, glare, photostress and visual perception [16,17,18,19,20,21,22,23,24,25,26,27,28,29,30,31,32,33,34,35,36,37,38,39,40,41,42,43,44,45,46,47,48]. Commercial terminology has evolved in parallel, with manufacturers applying differing definitions to “blue”, “blue–violet” and “HEV” light. This review therefore examines the evidence across modalities, identifies areas of convergence and uncertainty, and examines how closely commercial categories align with peer-reviewed findings. It is not intended to provide prescribing guidance.

## 2. Scope and Literature Approach

**Figure 1:**
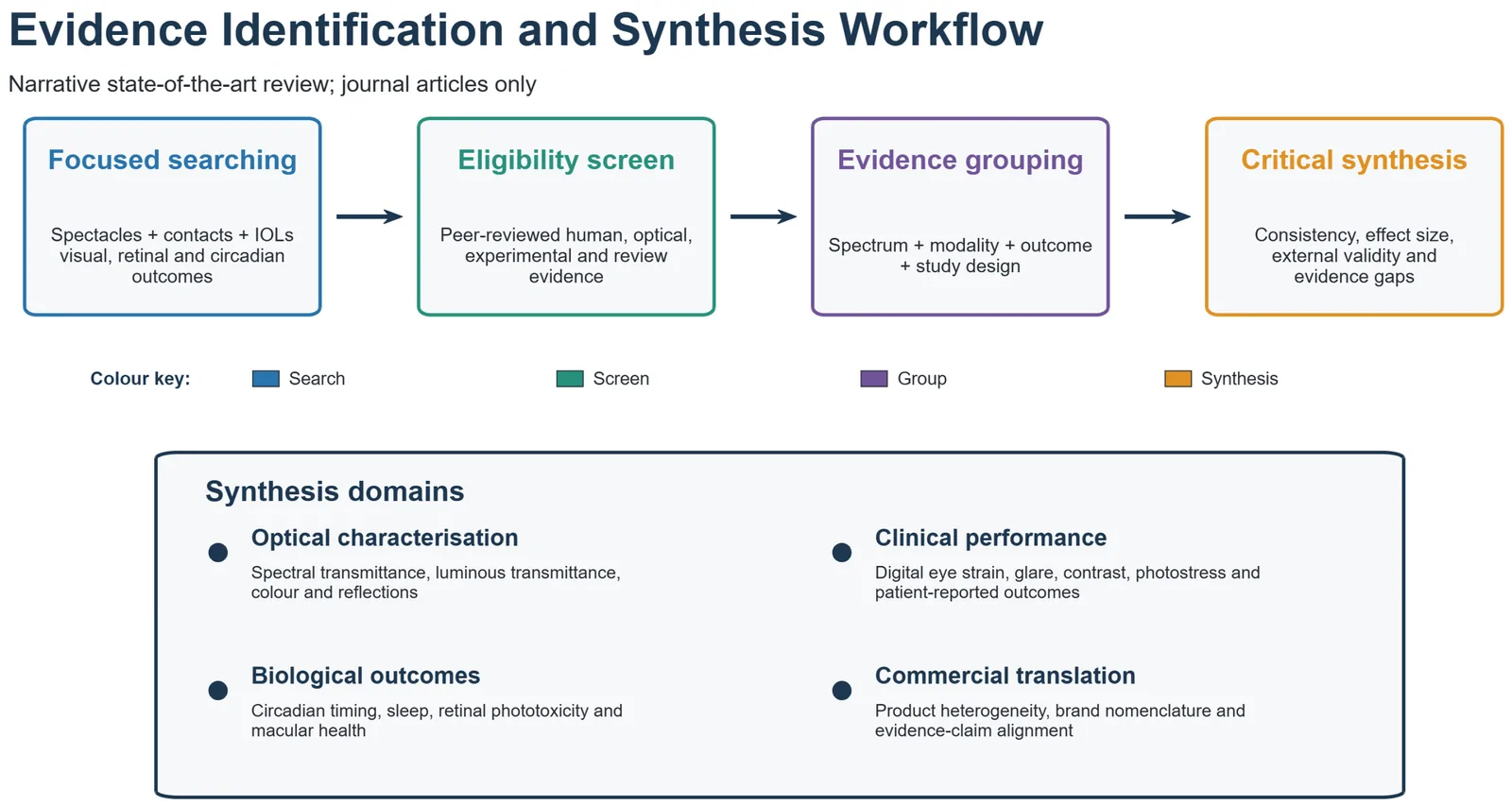
Evidence identification and synthesis workflow. The review uses a narrative state-of-the-art approach and organises journal evidence by modality, spectral characteristics, outcome domain and study design.

This article is a targeted narrative state-of-the-art review. Focused searches of PubMed/MEDLINE and reference lists were conducted for journal articles available up to 17 July 2026. Search concepts included blue light, blue–violet light, HEV light, spectral transmission, spectacle lenses, contact lenses, photochromic lenses, IOLs, digital eye strain, glare, photostress, colour, sleep, circadian rhythm, retina, retinal pigment epithelium, macular degeneration, adverse effects and tolerability. Systematic reviews, randomised and controlled studies, observational studies, optical characterisation studies and mechanistic experimental papers were considered. The synthesis deliberately incorporated positive, null and adverse findings, as well as optical trade-offs, funding context and methodological limitations. Conference abstracts, websites, patents and other non-journal material were excluded from the reference library. The resulting synthesis is qualitative and does not claim the exhaustiveness or formal risk-of-bias procedures of a registered systematic review. The evidence-identification and synthesis process is summarised in Figure 1. The searches were rechecked during revision to ensure inclusion of eligible journal articles published or available online by 17 July 2026. A formal score using the Scale for the Assessment of Narrative Review Articles (SANRA) was not applied prospectively. All figures in the current review were created using MATLAB R2026a (The MathWorks, Inc., Natick, MA, USA).

## 3. Terminology and Optical Characterisation

Visible blue light is commonly defined as approximately 400–500 nm, although commercial and scientific boundaries vary. The shorter “blue–violet” region is often distinguished from the blue–cyan range, which overlaps strongly with melanopsin sensitivity. Consequently, a statement that a lens “filters blue light” is incomplete unless accompanied by a spectral transmittance curve and a defined metric. Optical characterisation studies show substantial variation among nominally similar blue-light-filtering spectacle lenses in attenuation magnitude and wavelength selectivity [5,49,50,51]; coating-based lenses may also differ in surface reflectance [5].

**Figure 2:**
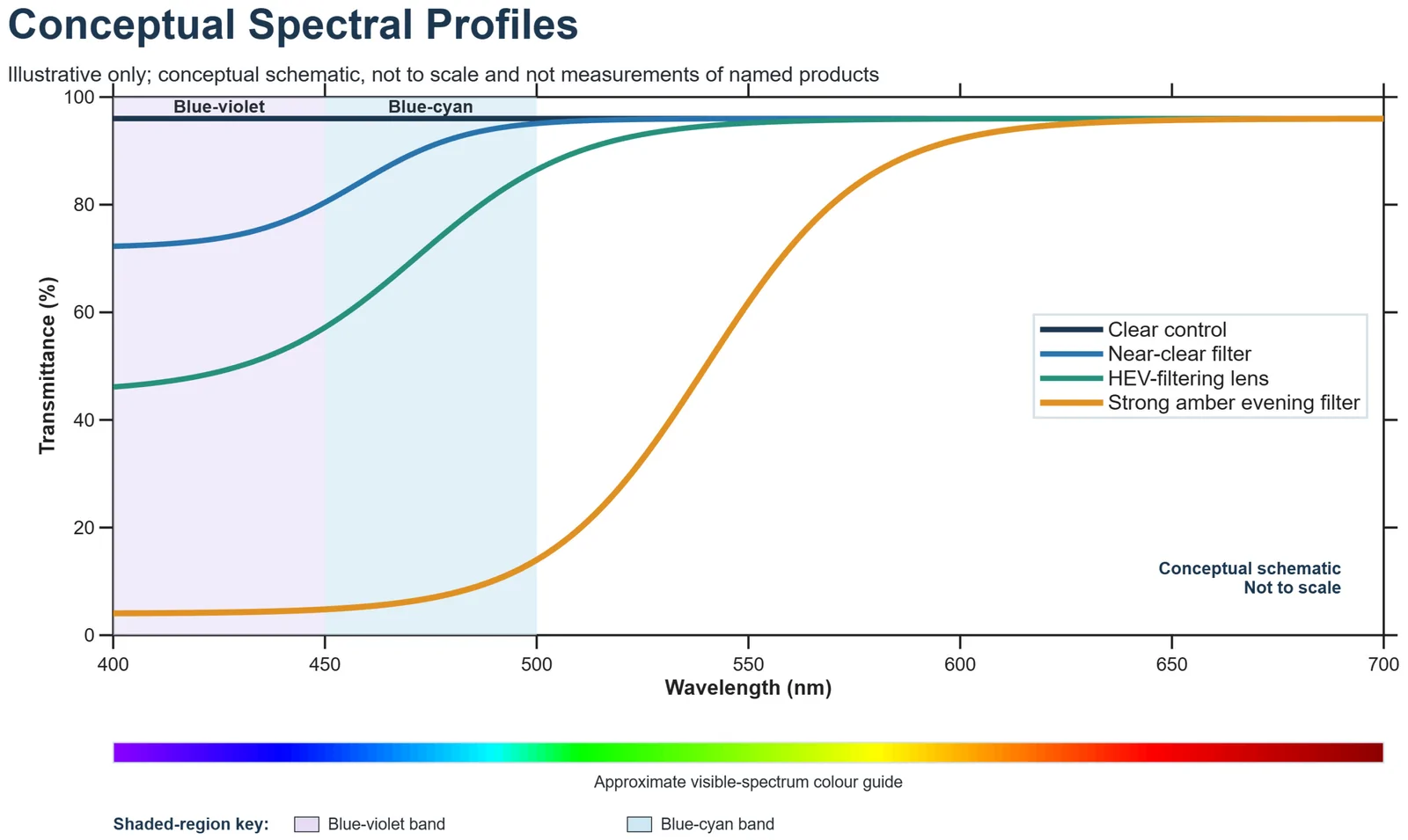
Conceptual spectral-transmittance profiles for clear control, near-clear spectacle filter, fixed HEV-filtering lens and strong amber evening filter. The curves are illustrative and do not represent measurements of named products. They synthesise the range of attenuation patterns reported in optical studies of spectacle and contact lenses [5,32,33,34,35,36,37,38,39,40,49,50,51]. The plotted curves are schematic and not to scale; the colour strip beneath the wavelength axis is an approximate visual guide to the visible spectrum and does not represent measured spectral data.

Four practical categories can be distinguished. Near-clear spectacle filters remove a modest proportion of selected short wavelengths while retaining high photopic transmittance. Strong amber, orange or red filters attenuate a broader range of short-wavelength visible light and can materially alter luminance and colour. Blue-filtering IOLs incorporate a chromophore within the implanted optic and remain in place continuously. Contact-lens technologies include light-adaptive photochromic materials and fixed HEV-filtering additives positioned directly on the eye. These categories should not be pooled without attention to their distinct optical properties and exposure schedules. These modality-specific distinctions are reflected in optical and clinical studies of spectacle lenses [5,49,50,51,52,53,54,55,56], IOLs [16,17,18,19,20,21,22,23,24,25,26,27,28,29,30,31,45,46] and contact lenses [32,33,34,35,36,37,38,39,40,43,44,47,48]. Figure 2 illustrates the contrasting spectral profiles, and Table 1 summarises the principal modalities, research outcomes and interpretive limitations.

## 4. Biological Context

### 4.1. Visual and Ocular-Surface Pathways

Digital eye strain encompasses ocular discomfort, dryness, burning, blurred vision, headache and musculoskeletal symptoms. Its recognised contributors include reduced blink rate, incomplete blinking, tear-film instability, uncorrected refractive error, accommodative and vergence demand, glare, workstation geometry and task duration [57,81,82,83,84,85]. Blue-light exposure is therefore only one proposed component of a multifactorial syndrome. A filter may alter retinal illuminance, scatter, chromatic cues or expectation without addressing the dominant ocular-surface or ergonomic mechanisms.

Optical effects vary by product. Near-clear filters usually have little effect on high-contrast acuity, while stronger filters may alter colour discrimination, contrast at selected spatial frequencies and mesopic performance. Short-term benefits in glare or photostress paradigms are plausible when a filter attenuates the spectral components that disproportionately contribute to intraocular scatter or discomfort. However, the magnitude and clinical relevance of these effects depend on the comparator and testing conditions [5,6,7,8,62,63,64,65,66,67].

### 4.2. Circadian and Neurobehavioural Pathways

Human circadian photoreception is particularly sensitive to short-wavelength light. Classic action-spectrum studies support a non-rod, non-cone photoreceptive pathway, and later work demonstrates wide interindividual variability [12,13,14,15]. Evening light from self-luminous displays can suppress melatonin, delay circadian timing and affect alertness under controlled exposure conditions [86,87,88,89,90]. The relevant exposure is not screen use alone, but melanopic retinal exposure integrated across spectrum, intensity, duration, timing, viewing distance, pupil size and concurrent ambient light.

A near-clear daytime spectacle filter may make only a small difference to melanopic stimulus, whereas a strongly attenuating amber filter worn for several hours before sleep can produce a much larger reduction. This difference explains why studies of psychiatric or sleep populations using dark evening filters should not be used to substantiate broad claims for lightly filtering clear lenses [52,53,54,55,56,70,71,72,73,74,75,76,77,78,79,80].

### 4.3. Photochemical and Retinal Pathways

Short-wavelength visible light can generate photochemical stress in experimental systems, particularly at high irradiance, after prolonged exposure or in models containing photosensitisers such as N-retinylidene-N-retinylethanolamine (A2E). Studies using cell and animal models have identified potential pathways involving reactive oxygen species, retinaldehyde photochemistry, inflammation and retinal pigment epithelium injury [91,92,93,94,95,96,97,98,99]. These findings establish biological plausibility but do not, by themselves, demonstrate harm from ordinary digital displays or show that consumer filters prevent AMD.

**Figure 3:**
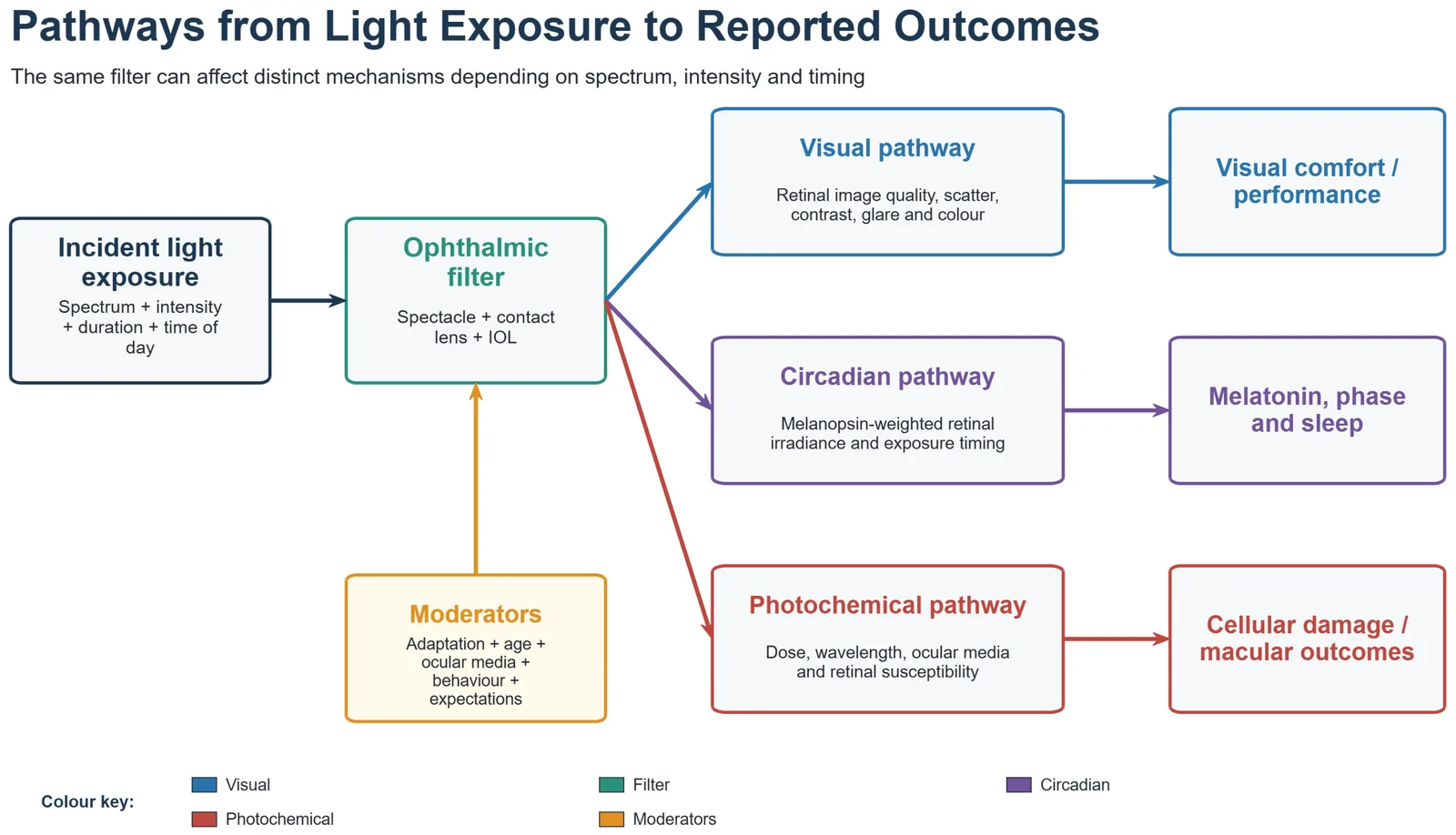
Visual, circadian and photochemical pathways linking incident light and ophthalmic filtration to clinical or biological outcomes. Although these pathways share an exposure source, they require different endpoints and should not be treated as a single therapeutic mechanism. The synthesis draws on visual, circadian and photochemical evidence [5,6,7,8,9,10,11,12,13,14,15,52,53,54,55,56,57,62,63,64,65,66,67,77,78,79,80,81,82,83,84,85,86,87,88,89,90,91,92,93,94,95,96,97,98,99].

These experimental findings must therefore be interpreted in relation to everyday exposure levels. Available assessments indicate that solid-state lighting and digital displays do not present an acute blue-light hazard under normal conditions of use [9,10]. Long-term epidemiological inference remains difficult because natural daylight contributes substantially more short-wavelength exposure than screens, while behaviour, cataract status, macular pigment, smoking, nutrition and genetic susceptibility are major potential confounders. Figure 3 summarises the distinct visual, circadian and photochemical pathways and their principal moderators.

### 4.4. Myopia and Short-Wavelength Exposure

Blue-light filtering should not currently be regarded as an established myopia-control intervention. The relevant literature is conceptually distinct because it has largely examined whether exposure to particular wavelengths, rather than filtration of routine blue light, can influence ocular growth. A two-year randomised trial of violet-light-transmitting spectacles in children did not show an unqualified overall treatment effect; reduced axial elongation was reported under selected subgroup conditions [100]. A 2024 systematic review and Bayesian network meta-analysis of wavelength-based interventions found that only red-light exposure produced a statistically significant pooled effect in the clinical comparisons, while evidence for violet light remained limited [101]. These findings should therefore not be extrapolated into a claim that commercial blue-light-filtering lenses prevent or slow childhood myopia.

## 5. Spectacle Lenses

### 5.1. Digital Eye Strain

Systematic reviews consistently report little or no short-term reduction in digital eye strain with blue-light-filtering spectacles [1,2,3,4,57]. The 2023 Cochrane review of 17 randomised trials found low-certainty evidence of little or no effect on subjective visual fatigue [1].

Trial-level evidence is largely consistent with the systematic reviews, although differences in interventions and study design may explain the occasional positive finding. Spectacle-lens trials found no symptom benefit [58,59], while other controlled studies reported little or no effect on visual symptoms, accommodative performance or electromyographic measures [60,67,69]. Positive findings have been reported in a small randomised trial [64], a ten-participant sham-controlled crossover pilot [71] and a cross-sectional questionnaire study [61]. However, differences in intervention, design and outcome measurement limit direct comparison. Overall, the evidence does not support blue-wavelength exposure as a primary cause of digital eye strain.

Although these findings do not support a class-wide therapeutic benefit, they do not imply that every wearer experiences every product in the same way. Subjective preference may be influenced by antireflection performance, tint, surface reflections, frame fit and individual light sensitivity. Such preferences should, however, be distinguished from demonstrated efficacy: a favourable response to a particular lens should not be extended to blue-light-filtering products as a class. This distinction is particularly important given the product variability and methodological challenges reported in optical studies, trials and reviews [1,5,58,59,60,68].

### 5.2. Visual Acuity, Contrast, Colour and Glare

Near-clear filters preserve standard photopic visual acuity in most studies, but laboratory studies identify small, product-specific changes in colour contrast, colour discrimination and contrast perception [5,6,7,8]. Stronger long-pass filters produce larger chromatic effects and may reduce mesopic or scotopic sensitivity. The practical importance depends on task demands: small shifts may be negligible for office work but relevant to colour-critical occupations, low-light driving or diagnostic colour interpretation.

Photostress and glare findings are mixed. Some studies report faster recovery or reduced symptoms under bright-light stress [62,63,64], whereas others show limited or condition-specific effects [65,66]. Comparisons can be confounded when test and control lenses differ in luminous transmittance, antireflection properties or visible tint. Spectral characterisation and photopic-equivalent comparators are therefore necessary before attributing an effect specifically to blue-wavelength attenuation [49,50,51].

### 5.3. Sleep and Circadian Effects

The sleep literature demonstrates stronger biological coherence than the digital-eye-strain literature, though its clinical interventions are highly heterogeneous. Controlled studies using evening amber or blue-blocking glasses report improvements in selected subjective or actigraphic outcomes across specific populations, including individuals with insomnia, delayed sleep phase disorder, mood disorders and athletes [53,54,72,73,74,75,76,77,79,80]. Notably, clinical trials in mania and bipolar disorder utilise blue-blocking lenses as a specialised, evening chronotherapeutic adjunct to deliberately restrict short-wavelength stimulation rather than as routine daytime eyewear [73,74,75].

Conversely, other trials report small, inconsistent or non-significant effects, especially in healthy populations or when spectral attenuation and participant adherence were modest [70,76,78]. In healthy adults, evening blue-blocking glasses did not improve objective sleep outcomes, and total sleep time showed a non-significant trend toward being shorter in the filtering condition [70]. Similarly, a clinical trial in pregnancy found no significant benefit for its primary diary or actigraphic sleep parameters, with transient side effects reported across both study groups [78].

Consequently, systematic reviews and meta-analyses describe mixed evidence. This inconsistency stems from a variable risk of bias and substantial methodological disparities in study populations, filter spectra, intervention timing and outcome measurements [52,55,56]. While evening spectral manipulation can successfully influence circadian physiology under optimised laboratory or clinical conditions, these therapeutic effects are neither uniform nor transferable to lightly filtering daytime lenses.

### 5.4. Reported Adverse Effects and Practical Trade-Offs

Potential disadvantages become more evident as spectral attenuation increases. Commercial near-clear filters can reduce colour contrast for blue stimuli, particularly under low contrast or low light, and lower-transmittance products induce more pronounced effects [6,7]. Greater short-wavelength attenuation can alter chromatic appearance and reduce retinal illuminance, but its effects on visual performance are not uniformly adverse. One study of two long-pass filter designs reported preserved photopic acuity and small improvements in mesopic acuity, visual acuity under glare and contrast sensitivity, which the authors attributed to reduced intraocular light scatter [66]. By comparison, a six-month randomised controlled trial of lenses providing approximately 15% and 30% blue-light attenuation found no clinically significant effect on photopic or scotopic contrast perception, including under glare conditions [8]. These findings indicate that visual effects are product-, population- and task-dependent.

Adverse-event reporting is sparse and inconsistent. In the Cochrane review, infrequent events reported in filtering-lens groups included headache, discomfort wearing the spectacles, lower mood and increased depressive symptoms; causality and comparative incidence were uncertain because studies were small and the certainty of evidence was low [1]. These observations do not establish common harm, but they show that tolerability, discontinuation and mood-related events should be reported rather than assumed absent.

### 5.5. Retinal and Macular Protection

No randomised clinical evidence demonstrates that blue-light-filtering spectacles prevent AMD or other chronic retinal disease [1,3,4]. Experimental retinal protection studies use conditions that are informative mechanistically but differ markedly from routine human exposure [91,92,93,94,95,96,97,98,99]. Claims of long-term retinal protection therefore remain broadly plausible at a biological level but clinically unproven for everyday clear spectacle filters. Established ultraviolet protection should also be separated from visible blue-light claims.

## 6. Blue-Filtering Intraocular Lenses

Blue-filtering IOLs form a distinct evidence domain because they are permanently implanted and replace the natural crystalline lens after cataract surgery. Comparative studies and reviews report similar high-contrast visual acuity with blue-filtering and ultraviolet-only IOLs, although findings for contrast and glare are more variable [16,17,18,19,20,21,22,23,24,25,26]. The evidence is not uniformly neutral, however. In a randomised fellow-eye study, eyes implanted with a blue-filtering IOL showed small but statistically significantly poorer contrast acuity and blue-yellow foveal thresholds than eyes implanted with a ultraviolet-only IOL. Three of 24 participants also reported a subjective difference in vision between the two eyes [41]. A separate comparative study found more blue-spectrum colour-discrimination errors under mesopic, but not photopic, illumination [42]. Other studies found no significant differences in scotopic sensitivity or hue discrimination [18], or in visual acuity, contrast sensitivity, colour perception and glare [25]. Systematic-review and meta-analytic evidence indicates broadly comparable visual acuity, contrast sensitivity and overall colour vision, while also identifying poorer blue-spectrum colour discrimination under mesopic conditions [16,23].

The proposed photoprotective rationale is that blue-light filtering IOLs reduce the amount of short-wavelength light reaching the retina after removal of the natural crystalline lens [17,26,29]. However, current evidence does not establish that this reduces the development or progression of macular disease. The 2018 Cochrane review found insufficient evidence to determine whether blue-filtering IOLs preserve macular health or alter AMD risk [16,30]; a large observational cohort found no apparent advantage for the incidence or progression of neovascular AMD [24]. More recent observational evidence remains mixed. In a 2024 cohort of eyes with neovascular AMD, blue-filtering IOLs were not associated with improved macular-atrophy-free survival. However, macular atrophy enlarged more slowly in the blue-filtering group [45]. A 2026 retrospective study with approximately three years of follow-up found no significant differences between blue-filtering and ultraviolet-filtering IOLs in retinal structural measures [46].

Non-visual benefits are similarly uncertain. A 2025 systematic review and meta-analysis found a slight, statistically non-significant improvement in subjective sleep quality, with no significant longer-term difference between blue-filtering and standard IOLs [31]. Theoretical work suggests that continuous short-wavelength attenuation may reduce scotopic and melanopsin-mediated photoreception [27,28], while clinical studies have reported mixed findings for scotopic and mesopic visual function [18,22,25,41,42]. Overall, neither macular protection [16,24,29,30] nor a clinically meaningful sleep benefit [31] has been established, and the clinical significance of reported mesopic and short-wavelength visual differences remains uncertain [18,22,25,41,42]. The 2026 study also found no significant between-group differences in self-reported sleep pattern or daytime alertness [46].

Future studies should report complete transmission spectra and account for IOL material, optic design, fellow-eye status, age, ambient lighting and baseline sleep characteristics.

## 7. Contact Lenses

### 7.1. Photochromic Contact Lenses

Photochromic contact lenses are relevant to blue-light filtration because their attenuation of short-wavelength light varies with activation. In the senofilcon A design, some filtration is present under minimally activated conditions, with greater attenuation following exposure to ultraviolet or violet light [37]. The optical effect is therefore continuous and environment-dependent rather than simply absent indoors and present outdoors.

Most evidence comes from short-term contralateral studies of this lens platform. Partial activation improved selected measures of photostress recovery, glare discomfort and disability, and chromatic contrast without affecting vernier acuity [32]. Related studies reported reductions in light scatter, halos and starbursts under both activated and minimally activated conditions [33,34,35]. This distinction is important: improvements observed in the minimally activated state cannot be attributed to outdoor darkening, while those observed after activation cannot be separated fully from the lens’s baseline spectral properties. The findings therefore support condition-specific effects of the complete photochromic lens system, but do not establish that increased blue-light attenuation alone produced the observed benefits.

Evidence from more routine visual tasks suggests that these effects do not come at the expense of standard visual performance. An indoor study reported improvements in kinetic and functional acuity and wearer satisfaction, but no significant differences in conventional visual acuity, contrast sensitivity or higher-order aberrations [36]. A separate simulation study found improved outdoor contrast sensitivity with the activated spectral profile, without a corresponding change in visual acuity [37]. In addition, a 24-participant randomised crossover study found daytime and night-time driving performance to be non-inferior to that with control lenses, with no adverse events reported during the study [43]. Collectively, these findings suggest possible benefits under bright-light or glare conditions while providing reassurance about short-term visual performance; they do not show that vision improves during routine wear.

The evidence remains limited by small samples, short follow-up and concentration on related iterations of a single commercial technology. Several studies were manufacturer-funded or involved manufacturer-affiliated authors [32,33,34,35,43]. Current evidence therefore supports selected short-term effects on glare, scatter and photostress, but does not establish benefits for digital eye strain, sleep, retinal protection or long-term ocular-surface health.

### 7.2. Fixed HEV-Filtering Contact Lenses

Fixed HEV-filtering contact lenses differ from photochromic designs because their selective short-wavelength attenuation is continuously present and does not depend on environmental activation. The available evidence is narrow, focusing primarily on short-term responses to intense light rather than routine visual symptoms or long-term clinical outcomes.

Two randomised, double-masked contralateral studies found that an HEV-filtering lens reduced halos, starbursts and behavioural measures of light scatter, while also reducing glare-related squinting and shortening photostress recovery time compared with a clear control lens [38,39]. These findings demonstrate that selective HEV attenuation can influence visual responses to bright broadband and violet light under controlled conditions. They do not, however, establish improved everyday visual performance or relief of symptoms during routine lens wear.

A subsequent randomised crossover study found that natural images appeared approximately 12% brighter through HEV-filtering lenses than through clear controls [40]. This result suggests that selective filtration can alter brightness perception rather than simply dimming the visual scene. Whether the change improves visibility, introduces a perceptual trade-off or has no meaningful functional effect remains uncertain because task performance was not assessed.

Evidence relating to electronic-device use is more limited. A prospective pilot study found between-lens differences in contrast sensitivity but no statistically significant improvement in tear-breakup time after display use with blue-filtering rather than standard contact lenses [44]. Because the study did not assess a validated symptom-based digital-eye-strain outcome, it does not establish relief of digital eye strain or an ocular-surface benefit.

**Figure 4:**
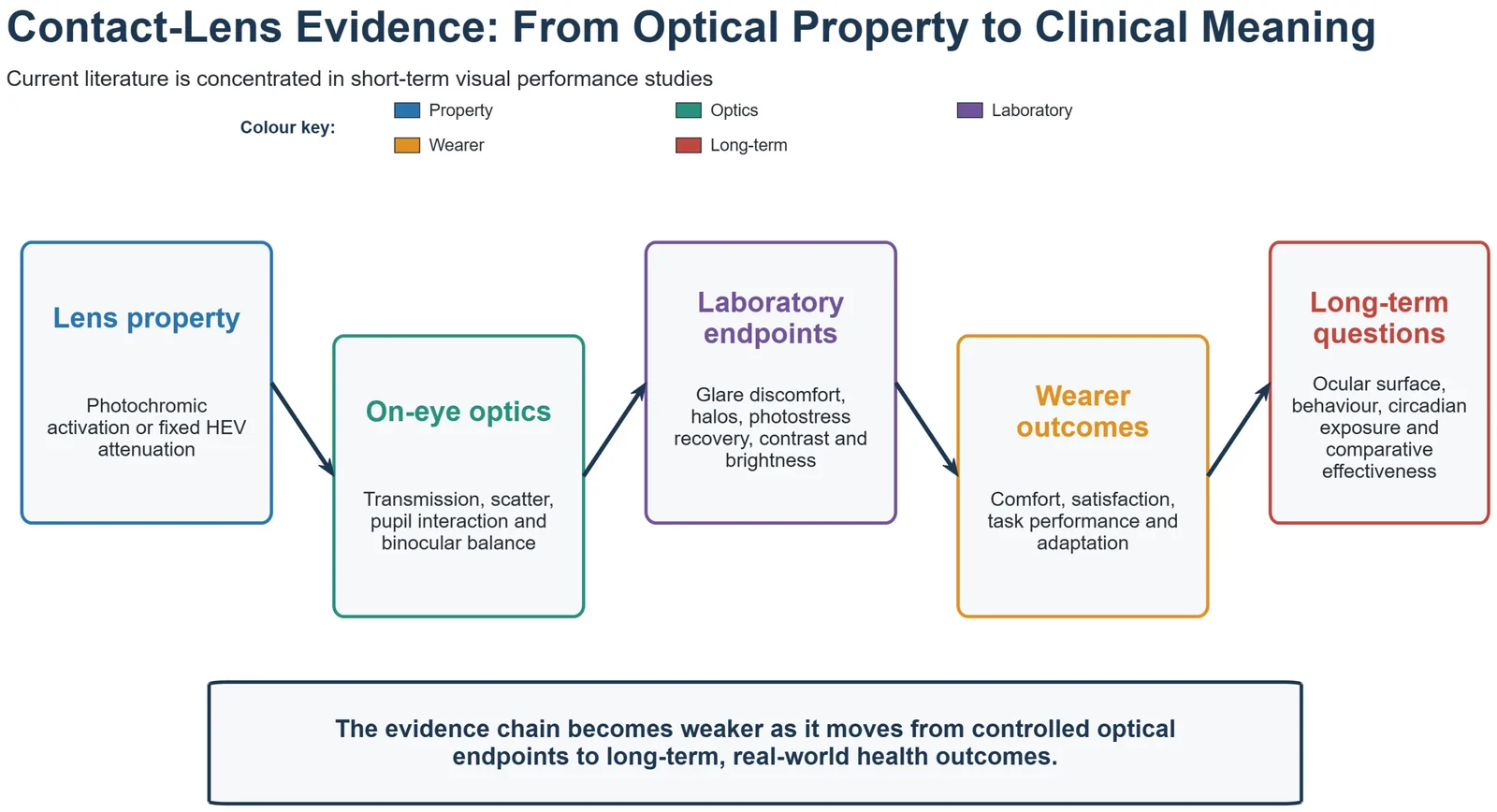
Contact-lens evidence pathway. Published research is strongest for optical properties and short-term laboratory visual endpoints, with progressively less evidence for everyday wearer outcomes, adverse effects and long-term health effects. The evidence gradient is based on the endpoints, durations and study designs reported in peer-reviewed contact-lens studies [32,33,34,35,36,37,38,39,40,43,44,47,48].

Overall, current evidence supports selected short-term effects on glare, photostress, scatter and brightness perception, but does not demonstrate broader real-world visual benefit. The identified studies do not establish benefits for digital eye strain, sleep, retinal protection or long-term ocular-surface health [38,39,40,44,47,48]. Interpretation is further limited by short-duration or non-dispensing designs and the concentration of the principal visual studies within one commercial technology; studies [38,39,40,47,48] also involved manufacturer-employed authors or manufacturer funding. Figure 4 illustrates this progression from laboratory optical outcomes to unresolved clinical questions, and Table 2 summarises the evidence and its limitations. Two studies published in 2026 before the search cut-off provide additional short-term evidence: one reported improved visual performance under simulated atmospheric blue-haze conditions, and another found higher photopic and mesopic contrast sensitivity with and without veiling glare [47,48]. Both studies involved manufacturer-employed or manufacturer-associated investigators and used controlled laboratory endpoints, so they do not resolve the need for independent real-world replication.

## 8. Commercial Landscape and Brand Nomenclature

Commercial products are best discussed in terms of their technology, measured spectrum and tested endpoint. Representative examples of current nomenclature include ZEISS BlueGuard, Essilor Crizal Prevencia, HOYA BlueControl and Nikon SeeCoat Next Blue for spectacle lenses, and ACUVUE OASYS MAX 1-Day for HEV-filtering soft contact lenses. Peer-reviewed optical studies have characterised some named products or predecessor generations, while contact-lens studies evaluate specific HEV-filtering and photochromic designs [5,32,33,34,35,36,37,38,39,40,43,44,47,48,49,50,51]. Interpretation should therefore be tied to the exact product generation, measured spectrum and outcome studied. The named products are non-exhaustive examples selected to represent the principal commercial technology categories for which relevant peer-reviewed optical or clinical literature was identified; inclusion does not imply comparative superiority or endorsement.

A recurring difficulty is that peer-reviewed studies may assess a predecessor product, laboratory sample, particular lens index or coating generation, or a product available only in selected markets. Formulations, metrics and claims can change faster than the clinical literature. Independent spectral surveys confirm substantial variation among commercial lenses marketed using similar blue-control terminology [50,51]. Evidence for a wavelength range or generic filter class should therefore not be presented as direct support for every current branded product.

**Figure 5:**
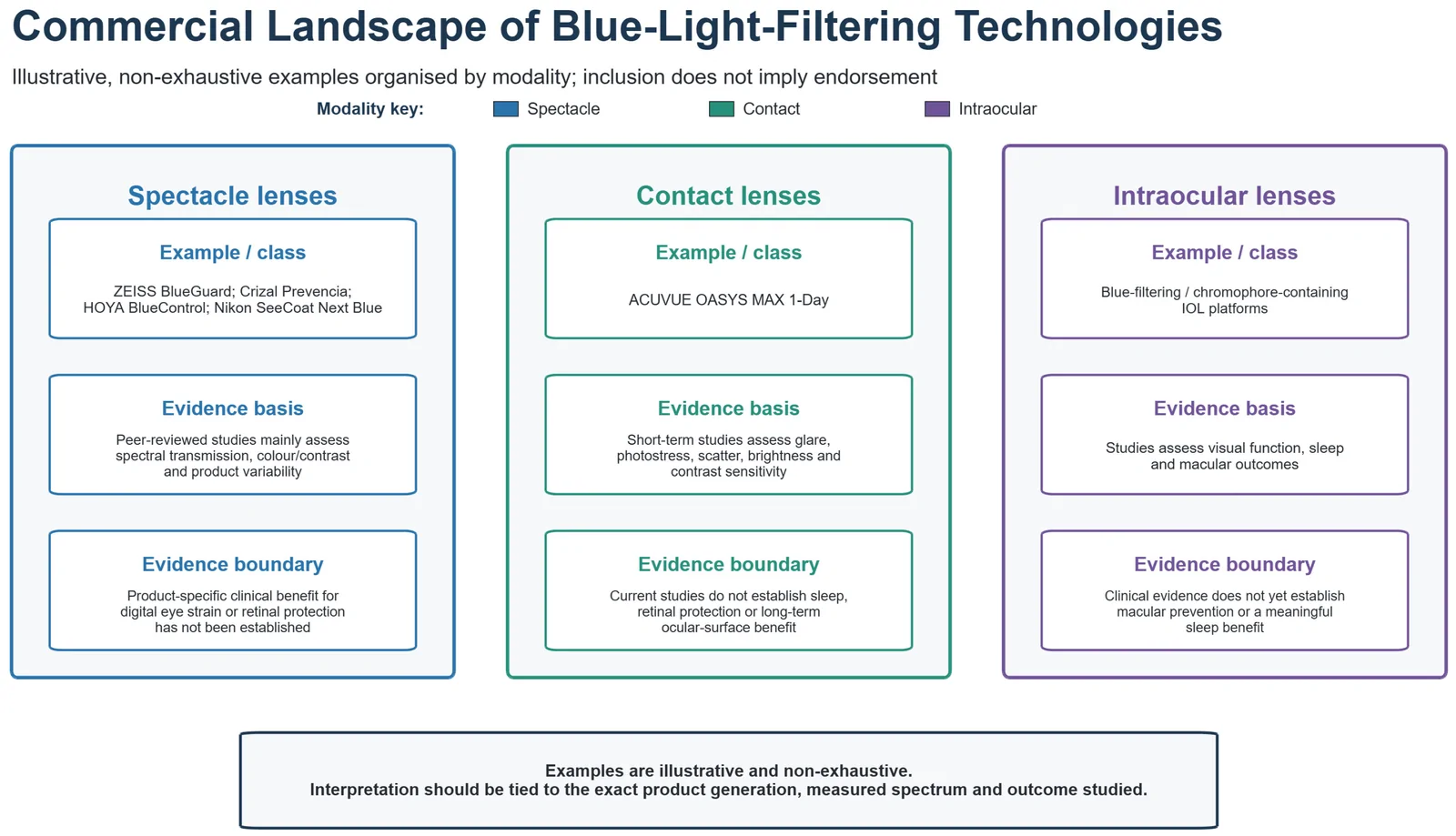
Commercial landscape of blue-light-filtering technologies. Representative, non-exhaustive examples are organised by modality; inclusion does not imply endorsement. Evidence basis/boundary references are: spectacles [5,49,50,51]/[1,2,3,4,5,57,58,59,60], contact lenses [38,39,40,47,48]/[38,39,40,44,47,48], and intraocular lenses [16,17,18,19,20,21,22,23,24,25,26,27,28,29,30,31,45,46]/[16,24,30,31,45,46].

Fair reporting requires three safeguards: manufacturer claims should be distinguished from independent clinical evidence; named products should be described using precise technological terms without extrapolating beyond tested outcomes; and relevant funding, consultancy or material support should be disclosed. Figure 5 separates each modality into an example or class, the evidence basis and the evidence boundary; Table 3 provides the corresponding product-level detail.

## 9. State-of-the-Art Synthesis

The central conclusion of this review is that blue-light filtering should not be treated as a single intervention. Interpretation depends on the spectral profile and magnitude of attenuation, luminous transmittance, activation state, wearing schedule, exposure conditions, comparator and outcome measured [1,5,16,50,51,52,56].

Within this framework, several conclusions are comparatively stable. Near-clear spectacle lenses provide little or no average short-term reduction in digital eye strain [1,2,3,4,57,58,59,67]. Experimental studies support the biological plausibility of short-wavelength photochemical effects [91,92,93,94,95,96,97,98,99], but assessments of normal consumer lighting and digital-display use do not indicate an acute blue-light hazard [9,10], and no clinical evidence shows that consumer spectacle filters prevent retinal disease [1,3,4]. Substantial reduction in evening short-wavelength exposure can alter circadian physiology [12,13,14,15,86,87,88,89,90], but effects on sleep remain heterogeneous and depend on attenuation, timing, adherence, population and ambient light exposure [52,53,54,55,56,70,72,73,74,75,76,77,78,79,80]. Greater attenuation may also alter colour and luminance [6,7,66], while adverse events have been reported inconsistently and remain poorly characterised [1].

Evidence for glare, photostress and visual perception is more context-dependent. Favourable task-specific effects have been reported with spectacle lenses [62,63,64,65,66], an IOL under glare-related driving conditions [19], and photochromic or fixed HEV-filtering contact lenses [32,33,34,35,36,37,38,39,47,48]. These findings arise from different spectra, comparators, illumination conditions and endpoints and should not be interpreted as evidence of broad improvement in visual performance. For IOLs, high-contrast acuity and other conventional visual outcomes are broadly comparable between blue-filtering and ultraviolet-only designs [16,18,20,23,25,26], although individual studies have reported small contrast or blue-spectrum disadvantages, particularly under mesopic conditions [41,42]. Contact-lens evidence is strongest for short-term visual-performance, glare, scatter, photostress and brightness outcomes [32,33,34,35,36,37,38,39,40,43,47,48]. It remains insufficient for validated digital-eye-strain symptoms, sleep, retinal protection or long-term ocular-surface outcomes [32,33,34,35,36,37,38,39,40,43,44,47,48], and many of the principal visual studies were manufacturer-associated [32,33,34,35,38,39,40,43,47,48].

**Figure 6:**
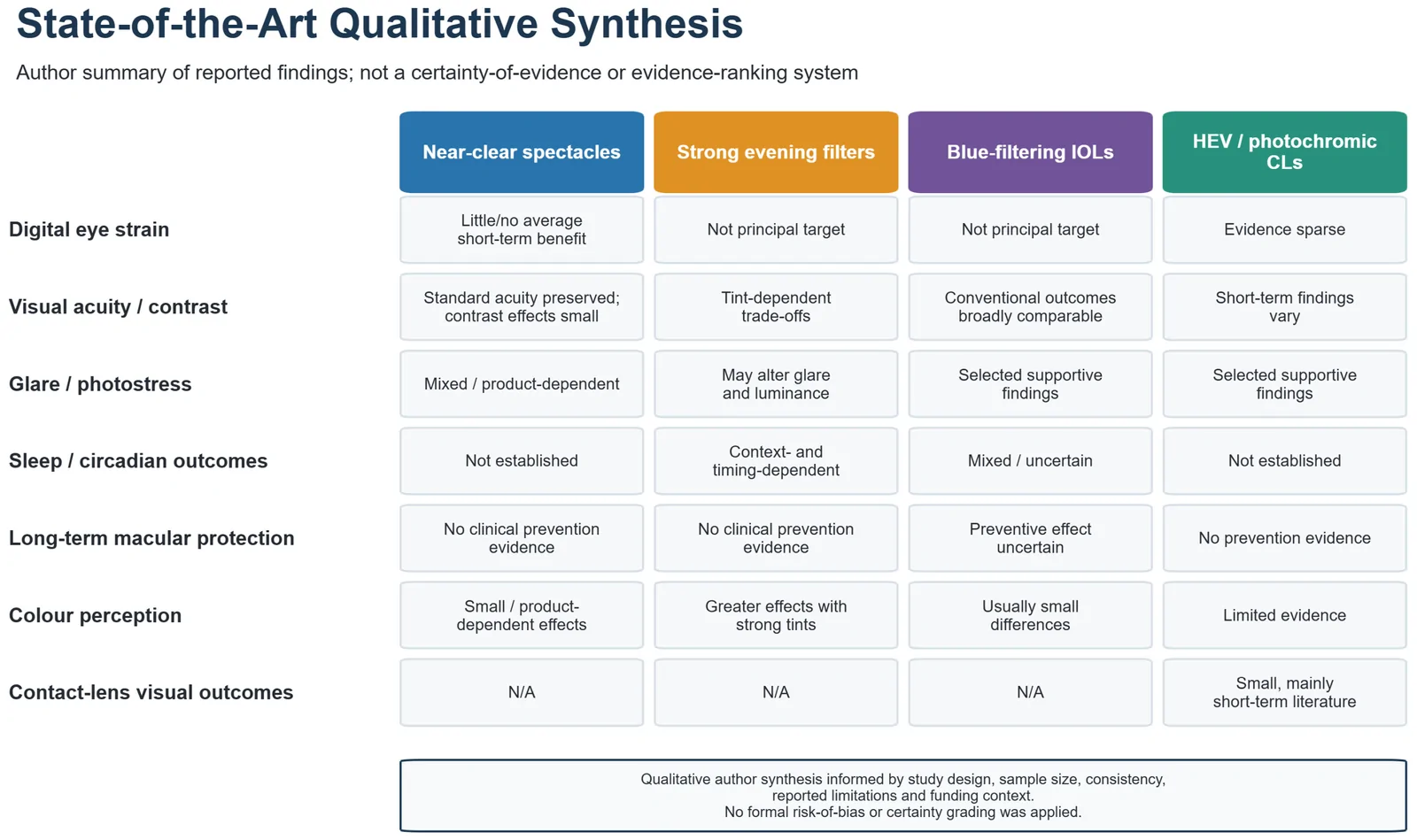
State-of-the-art qualitative synthesis across the principal blue-light-filtering modalities and claimed outcomes. The figure summarises reported findings and trade-offs in neutral cells and is not an evidence-ranking framework or a formal certainty-of-evidence assessment. The wording reflects the considerations described in Section 2 rather than a numerical score. The synthesis is based on systematic reviews and representative optical and clinical studies [1,2,3,4,5,6,7,8,16,17,18,19,20,21,22,23,24,25,26,27,28,29,30,31,32,33,34,35,36,37,38,39,40,41,42,43,44,45,46,47,48,49,50,51,52,53,54,55,56,57,58,59,60,61,62,63,64,65,66,67,68,69,70,71,72,73,74,75,76,77,78,79,80,86,87,88,89,90,91,92,93,94,95,96,97,98,99].

**Figure 7:**
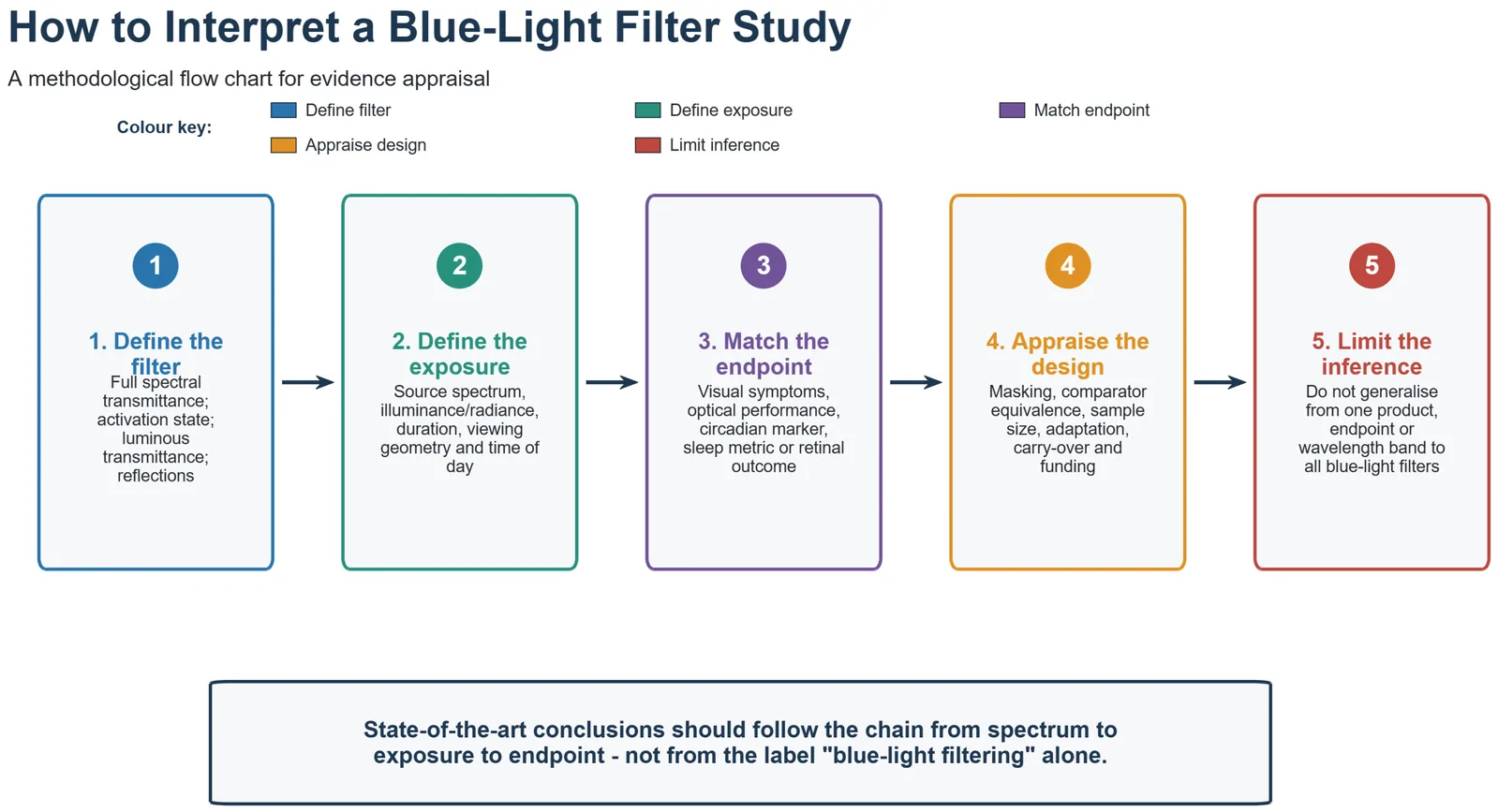
Framework for appraising studies of blue-light filtration. Valid inference requires alignment among the measured filter spectrum, activation state, luminous transmittance, exposure conditions, comparator, endpoint and study design. The framework reflects recurring methodological limitations identified in systematic reviews and optical-characterisation studies [1,5,16,50,51,52,55,56].

Commercial translation has outpaced independent comparative evidence. Products marketed under similar terminology can differ substantially in spectral transmission, and published findings may relate to a specific lens material, coating, index, product generation or regional formulation [5,49,50,51]. Evidence should therefore be linked to the exact product tested, its measured spectrum, the exposure conditions and the endpoint assessed. Mechanistic plausibility should not be presented as demonstrated clinical efficacy, and findings from one technology or product class should not be extrapolated to every current branded product. Figure 6 provides a qualitative synthesis across modalities and outcomes. The wording in Figure 6 was developed as an author summary, considering the direction and consistency of findings, study design, sample size, duration, risk-of-bias concerns reported in systematic reviews, and manufacturer involvement; no numerical score or predefined certainty threshold was used. Figure 7 sets out a framework for appraising individual studies, and Table 4 summarises the evidence by claimed outcome.

## 10. Research Gaps

Future studies should begin with adequate optical characterisation. Complete spectral transmittance should be reported in the worn or activated state, together with luminous transmittance, surface reflectance and colour coordinates where applicable. Photopic and melanopic quantities should be provided when visual or circadian effects are claimed. Reports should identify the exact product generation, material, coating, lens index and activation conditions. Comparator lenses should be matched as closely as possible for prescription, lens design, antireflection properties, visible tint and luminous transmittance. Without this information, effects cannot be attributed specifically to short-wavelength attenuation.

Clinical studies should be preregistered, adequately masked and powered to detect prespecified clinically meaningful differences. Outcomes should be validated and appropriate to the claim being tested, and follow-up should be long enough to distinguish transient laboratory effects from sustained clinical benefit. Adverse events, discontinuations, colour-related complaints, mood effects and tolerability should be collected prospectively rather than reported incidentally.

Research priorities differ by modality. Digital-eye-strain trials should assess validated symptoms alongside blinking, tear-film function, refractive status, accommodation, vergence, task duration and ergonomics. Sleep studies should quantify the spectrum and intensity of ambient and device light, intervention timing, adherence and objective circadian or sleep outcomes. IOL studies require adequate power and long follow-up for macular outcomes, together with standardised mesopic testing and control for age, ocular comorbidity, IOL material and optic design. Contact-lens studies should include independent replication, real-world wear, adaptation, ocular-surface safety and direct comparisons among photochromic, fixed HEV-filtering and clear designs.

Commercially relevant research should report the exact product generation, funding source, investigator relationships and data ownership. Independent head-to-head comparisons of current products would be valuable, but their interpretation must remain anchored to measured spectra and clinically meaningful endpoints rather than marketing categories.

## 11. Conclusions

Blue-light filtering in eye care encompasses optically and clinically distinct spectacle, contact-lens and intraocular technologies. Near-clear spectacle filters do not provide a clinically meaningful average reduction in short-term digital eye strain, and clinical prevention of retinal disease by consumer spectacle or contact-lens filters has not been demonstrated. Substantial evening attenuation can modify circadian exposure, but sleep benefits are inconsistent and should not be extrapolated to lightly filtering daytime lenses. Conventional visual function is preserved in most studies of blue-filtering IOLs, although small mesopic and short-wavelength differences have been reported and macular and sleep benefits remain uncertain. Contact-lens studies show selected short-term effects on glare, scatter, photostress and brightness perception, but do not establish broader or long-term clinical benefit. Greater attenuation may introduce colour and luminance trade-offs, while adverse-event reporting remains inadequate.

The state of the art is therefore not defined by a single verdict on “blue-light filters”. Each claim must instead be evaluated against the exact spectrum, modality, exposure conditions and outcome studied. Progress will depend on independent, spectrally characterised comparisons using clinically meaningful endpoints.

## Figures and Tables

**Table 1:** Principal blue-light-filtering modalities in contemporary eye care.

Modality	Typical Optical Behaviour	Common Research Outcomes	Key Interpretive Issue
Near-clear spectacle lens	Selective, modest short-wavelength attenuation with high luminous transmittance [5,49,50,51]	Digital eye strain, colour, contrast and glare [1,2,3,4,5,6,7,8,50,57,58,59,60,61,62,63,64,65,66,67,68,69]	Products with similar appearance can have materially different spectra [5,49,50,51]
Strong tinted spectacle filter	Broad short-wavelength attenuation, commonly accompanied by lower luminous transmittance and greater colour change [52,53,54,55,56,70,71,72,73,74,75,76,77,78,79,80]	Melatonin suppression, circadian phase and sleep [52,53,54,55,56,70,71,72,73,74,75,76,77,78,79,80]	Substantial attenuation can reduce luminous transmittance and alter colour; effects depend on spectrum and task [1,6,7,52,66,70,78]
Blue-filtering IOL	Continuous intraocular short-wavelength filtration after cataract surgery [16,17,18,19,20,21,22,23,24,25,26,27,28,29,30,31]	Visual function, sleep and macular health [16,17,18,19,20,21,22,23,24,25,26,27,28,29,30,31]. Recent retinal and self-reported functional outcomes are also represented [45,46].	Permanent filtration; small mesopic or short-wave disadvantages have been reported alongside broadly comparable outcomes [16,17,18,19,20,21,22,23,24,25,26,27,28,29,30,31,41,42]
Photochromic contact lens	Transmission varies with activation state and environmental conditions [32,33,34,35,36,37]	Glare, scatter, dysphotopsia and short-term visual performance [32,33,34,35,36,37]	Activation and performance depend on environment; evidence is short-term and often manufacturer-associated [32,33,34,35,36,37,43]
Fixed HEV-filtering contact lens	Stable selective attenuation incorporated into a soft contact-lens design [38,39,40]	Glare, photostress recovery, brightness and visual perception [38,39,40]. Recent studies also assess contrast sensitivity and blue-haze visual performance [47,48].	The literature is small, short-term and technology-specific, with limited independent and ocular-surface evidence [38,39,40,44,47,48]

HEV, high-energy visible; IOL, intraocular lens. Categories are descriptive rather than regulatory.

**Table 2:** Selected peer-reviewed contact-lens studies relevant to blue-light and HEV filtration.

Technology	Representative Design	Principal Endpoints	State-of-the-Art Interpretation
Photochromic soft lens	Contralateral, randomised or crossover comparisons [32,33,34,35,36,37,43]	Glare discomfort, photostress, scatter, dysphotopsia and indoor visual performance [32,33,34,35,36,37]	Short-term visual effects are plausible; small samples, surrogate endpoints and frequent manufacturer involvement limit wider applicability [32,33,34,35,36,37,43]
Fixed HEV-filtering soft lens	Double-masked contralateral studies [38,39]. Later bilateral crossover/contrast-sensitivity studies were also identified [47,48].	Halos, starbursts, glare discomfort and photostress recovery [38,39]. Recent endpoints include contrast sensitivity under blue-haze and veiling-glare conditions [47,48].	Early supportive laboratory evidence; independent replication and longer real-world follow-up are needed [38,39]. The newer studies remain short-term and manufacturer-associated [47,48].
Fixed HEV-filtering soft lens	Natural-image brightness study [40]	Perceived brightness and image appearance [40]	HEV filtration changes perceived brightness, but its functional significance and any perceptual trade-off remain uncertain [40]
Across contact-lens technologies	The identified literature is small and mainly of short duration; several studies are manufacturer-associated [32,33,34,35,36,37,38,39,40,43,44,47,48]	Optical and visual-performance outcomes dominate; one pilot found no significant tear-breakup advantage [32,33,34,35,36,37,38,39,40,43,44,47,48]	Evidence on long-term ocular-surface, sleep, disease-prevention, adverse-event and comparative-effectiveness is limited [32,33,34,35,36,37,38,39,40,43,44,47,48]

The table summarises the reported evidence domains and their limitations.

**Table 3:** Evidence basis and boundaries for selected commercial technologies.

Example/Class	Modality/Approach	Evidence Basis	Evidence Boundary
ZEISS BlueGuard	Spectacle lens; commercial in-material blue-light filtering.	Peer-reviewed studies mainly assess spectral transmission, colour/contrast and product variability [5,49,50,51].	Product-specific clinical benefit for digital eye strain or retinal protection has not been established [1,2,3,4,5,57,58,59,60].
Crizal Prevencia/HOYA BlueControl/Nikon SeeCoat Next Blue	Spectacle-lens coatings or selective-filtering products; named or predecessor versions have undergone optical characterisation [5,49,50,51].	Peer-reviewed studies mainly assess spectral transmission, colour/contrast and product variability [5,49,50,51].	Evidence does not establish product-specific clinical benefit for digital eye strain or retinal protection; independent head-to-head comparisons of current products remain limited [1,2,3,4,5,49,50,51,57,58,59,60].
ACUVUE OASYS MAX 1-Day	Fixed HEV-filtering soft contact lens.	Short-term studies assess glare, photostress, scatter, brightness and contrast-sensitivity outcomes [38,39,40,47,48].	These studies do not establish sleep benefit, retinal protection or long-term ocular-surface benefit [38,39,40,44,47,48].
Blue-filtering IOL platforms	Chromophore-containing implanted optics.	Studies assess visual function, sleep and macular outcomes, including recent observational retinal and self-reported outcomes [16,17,18,19,20,21,22,23,24,25,26,27,28,29,30,31,45,46].	Clinical evidence does not yet establish macular prevention or a meaningful sleep benefit; spectra and materials are not interchangeable [16,24,30,31,45,46].

Product names are used descriptively. Trademark ownership remains with the respective manufacturers.

**Table 4:** Summary of benefits, null findings and reported disadvantages by claimed outcome.

Claimed Outcome	Spectacle Lenses	IOLs	Contact Lenses	Overall State
Reduction in digital eye strain	Reviews and controlled trials show little or no average short-term symptom benefit [1,2,3,4,57,58,59,67].	Not a principal outcome in the reviewed IOL literature [16,17,18,19,20,21,22,23,24,25,26,27,28,29,30,31].	Not established. One pilot assessed tear-film and contrast outcomes during device use rather than validated symptom relief [44].	No benefit has been established for blue-light filtering as a class; the available symptom evidence relates predominantly to spectacle lenses [1,2,3,4,44,57,58,59,67].
Improved glare or photostress	Findings are mixed and product- and task-specific [62,63,64,65,66].	Supportive evidence is limited to selected glare-related tasks [19].	Short-term improvements have been reported under controlled glare and bright-light conditions [32,33,34,35,36,37,38,39,47,48].	Effects are plausible under selected conditions but do not establish broad improvement in visual performance [19,32,33,34,35,36,37,38,39,47,48,62,63,64,65,66].
Improved sleep or circadian timing	The biological rationale is strongest for substantial evening attenuation, but clinical sleep outcomes are mixed [52,53,54,55,56,70,72,73,74,75,76,77,78,79,80].	Effects are small and imprecise, with no established clinically meaningful or durable benefit [31]. A 2026 observational study likewise found no difference in self-reported sleep pattern or daytime alertness [46].	Not evaluated in the identified studies [32,33,34,35,36,37,38,39,40,43,44,47,48].	Effects depend on spectrum, timing, exposure, adherence and population and cannot be transferred across filter types [31,52,55,56].
Retinal or macular protection	No clinical prevention evidence exists for consumer spectacle filters [1,3,4]; assessments of normal consumer sources do not indicate an acute blue-light hazard [9,10].	It remains unclear whether blue-filtering IOLs preserve macular health or alter AMD risk; observational evidence has not shown a clear preventive advantage [16,24,30]. Recent observational work found no reduction in new-onset macular atrophy or retinal structural difference, although one nAMD cohort reported slower macular-atrophy enlargement [45,46].	No clinical disease-prevention studies were identified [32,33,34,35,36,37,38,39,40,43,44,47,48].	Experimental plausibility exceeds demonstrated clinical prevention evidence [1,3,4,16,24,30,45,46,91,92,93,94,95,96,97,98,99].
Preservation or improvement of visual function	High-contrast acuity is preserved in most studies, although stronger filters can affect colour discrimination and low-light performance [5,6,7,8,66].	Conventional outcomes are broadly comparable, but small mesopic or blue-spectrum differences occur in some studies [16,18,20,23,25,26,41,42].	Standard acuity is preserved in short-term studies; glare, scatter, contrast and brightness can vary with filter design and activation [32,33,34,35,36,37,38,39,40,43,44]. Additional 2026 studies report short-term contrast-sensitivity benefits under controlled blue-haze or glare conditions [47,48].	Effects depend on spectrum, luminous transmittance, comparator, adaptation and task [5,6,7,8,16,18,20,23,25,26,32,33,34,35,36,37,38,39,40,41,42,43,44,47,48,66].
Trade-offs and adverse effects	Greater attenuation can alter colour and luminance. Headache, spectacle discomfort and mood-related events have been reported infrequently and inconsistently [1,6,7,66].	Some studies report small mesopic or short-wavelength disadvantages, while theoretical concerns remain regarding scotopic and circadian photoreception [27,28,41,42].	Long-term filter-specific safety has not been established. Most studies were short, and one pilot found no significant tear-film benefit [32,33,34,35,36,37,38,39,40,43,44,47,48].	Published studies have not identified a consistent safety signal, but adverse-event ascertainment and long-term follow-up remain inadequate [1,16,43,44].

The synthesis reflects evidence available to 17 July 2026.

## Data Availability

No new data were created or analysed in this study. Data sharing is not applicable to this article.

## Abbreviations

AMD	age-related macular degeneration
HEV	high-energy visible
IOL	intraocular lens
A2E	N-retinylidene-N-retinylethanolamine
SANRA	Scale for the Assessment of Narrative Review Articles
